# Virtual Screening and Bioassay of Novel Protoporphyrinogen Oxidase and *p*-Hydroxyphenylpyruvate Dioxygenase Dual-Target Inhibitors

**DOI:** 10.3390/molecules30071491

**Published:** 2025-03-27

**Authors:** Panxiu Zhang, Haifeng Cao, Tiansong Li, Ying Fu

**Affiliations:** 1 Department of Chemistry, College of Arts and Sciences, Northeast Agricultural University, Harbin 150030, China; zhangpanxiu2022@163.com (P.Z.); caohf@neau.edu.cn (H.C.); s231101011@neau.edu.cn (T.L.); 2 National Soybean Engineering Technology Research Center, Northeast Agricultural University, Harbin 150030, China; 3 Key Laboratory of Agricultural Functional Molecule Design and Utilization of Heilongjiang Province, Harbin 150030, China

**Keywords:** HPPD, PPO, dual-target herbicide, skeleton-based drug discovery, Bayesian model

## Abstract

Novel herbicide development is a challenge for weed control. Protoporphyrinogen oxidase (PPO) and *p*-hydroxyphenylpyruvate dioxygenase (HPPD) are two key enzymes involved in plant photosynthesis. The multi virtual screening protocol was adopted to design a common skeleton based on the two target enzymes, and fragment growth of the skeleton was performed. The constructed compounds were searched for structural similarity, and the accuracy of the selected compounds was further verified using the Bayesian model. Finally, eight compounds were obtained, and the binding mode with the target was studied deeply. The obtained compounds interact with the key residues of HPPD and PPO proteins similarly to commercial herbicides, and the stability of binding with proteins is also good. The activity of the screening results was determined by an enzyme activity test in vitro. The herbicidal effect of the compound was studied by phenotypic experiment. The final results showed that Z-4 and Z-7 have the potential to become new dual-target herbicides.

## 1. Introduction

Crop growth is disturbed by weeds competing with crops for water, sunlight, and other resources. Overuse of herbicides leads to weed resistance and causes environmental problems [1,2]. The development of environmentally friendly pesticides is one of the effective means to manage resistant weeds [3]. Chlorophyll and carotenoid play a key role in photosynthesis [4]. Chlorophyll is the pigment required by plants to absorb light energy. Carotenoid plays a protective role and avoids single-line oxygen damage to the photosystem. *P*-Hydroxyphenylpyruvate dioxygenase (HPPD) and protoporphyrinogen oxidase (PPO) are two key oxidases in the synthesis of photosynthetic pigments. In plants, HPPD catalyzes the conversion of *p*-hydroxyphenylpyruvate (HPPA) to homogentisic acid (HGA), which further reacts to produce plastoquinone and tocopherol [5,6,7]; plastoquinone is a key cofactor in carotenoid synthesis. HPPD inhibitors hinder the formation of plastoquinone and pigment [8]. PPO plays a key role in chlorophyll biosynthesis. Under aerobic conditions, PPO catalyzes the oxidation of protoporphyrinogen IX to protoporphyrin IX [9]. Protoporphyrinogen IX is accumulated in the cytoplasm if PPO is inhibited; under aerobic conditions, it is further oxidized to protoporphyrin IX, which leads to the blockage of chlorophyll synthesis [10,11]. HPPD and PPO inhibitors interfere with plants’ photosynthesis by inhibiting the synthesis of photosynthetic pigments.

Dual-target inhibitors, which simultaneously target two targets, provide different effects than single-target inhibitors, including a lower resistance risk and lower dosage [12]. Drug design based on dual targets has attracted wide attention in drug development. In albino herbicide design, complementary ligands were used to construct pharmacophore models, and the optimal models were used to screen commercial databases. The selected compounds were further simulated to find dual-target inhibitors of HPPD and phytoene desaturase (PDS) [13]. This method provides a promising lead for the design of new dual-target herbicides.

Computer-aided drug design (CADD) has become a common method in drug development [14]. Pharmacophore screening, molecular docking, molecular dynamics (MD) simulation, toxicological prediction, and other related approaches have been widely examined in drug development. With the development of artificial intelligence (AI), AI has been extensively applied in new drug design and has achieved inspiring results, which accelerates the process of new drug development and reduces the cost of drug development [15]. Machine learning (ML) and deep learning models improve screening hit rates and accuracy [16,17]. Varieties of ML models (k-Nearest Neighbor, support vector machines, Bayesian models, random forests, and neural networks) have been applied in drug design [14]. For example, ML models were used to discover novel estrogen receptor (ER) agonists. Random forest and deep neural network models were constructed based on the extended connectivity fingerprint (ECFP) of compounds; 10 compounds were selected for further evaluation, and finally, 7 compounds previously unreported were confirmed as ER agonists [18]. The combination of ML and traditional drug design provides new insights into drug development.

**Figure 1:**
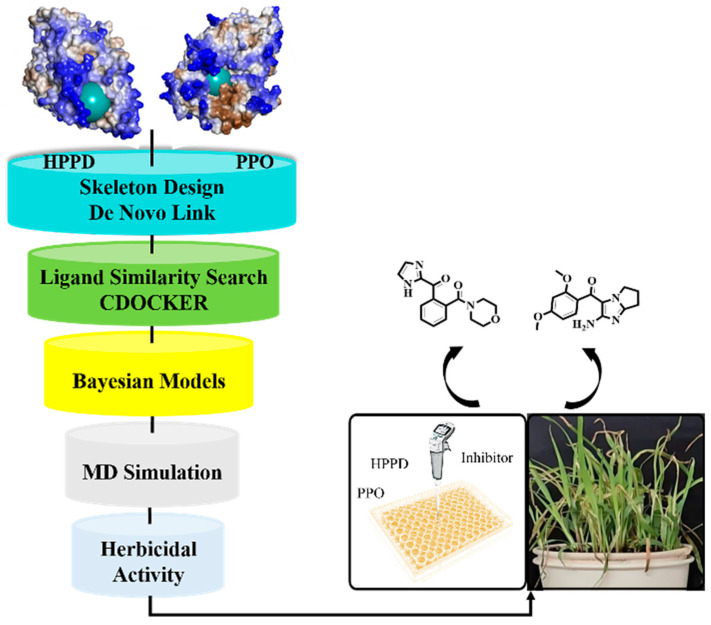
The workflow of multi virtual screening.

In this study, skeleton growth, combined with ligand similarity search techniques, was employed to design potential inhibitors for HPPD and PPO. A total of five compounds with good effects were constructed and selected for similar ligand screening in the database (about 280,000 compounds), and 158 obtained compounds were sent for molecular docking. ML was selected to construct a Naïve Bayesian Classification model to further improve the accuracy and efficiency of HPPD and PPO inhibitor screening, and finally eight potential compounds were obtained. The workflow for the searching of HPPD and PPO dual-target inhibitors is shown in Figure 1.

## 2. Results and Discussion

### 2.1. Screening Based on Fragment Library and Molecular Docking

The De Novo Link function in DS was selected to accomplish fragment screening. A total of 481 compounds were generated during fragment library screening for HPPD inhibitors, and 368 compounds were generated for PPO. In total, 25 intersecting molecules were generated, the physical and chemical properties of which were predicted (Table S1); all compounds conformed to the Lipinski principle (MW ≤ 500, HBD < 5, HBA < 10, Log *p* < 5, RB ≤ 10). The 25 compounds were sent to the molecular docking procedure with two targets, respectively. According to the interaction with residues, for the HPPD target, the compounds formed interactions with residues Phe424, Phe381, Phe392, Phe419, Gln293, and His308 in protein. Residues Phe424 and Phe381 mainly produced π-π interactions with ligands, residue His308 produced hydrogen bond interactions, and Phe392 produced hydrophobic interactions (Table S2). For the PPO target enzyme, the keys residues were Phe392, Arg98, Leu372, and Leu356. Arg98 mainly produced hydrogen bond interactions, Leu372 and Leu356 produced hydrophobic interactions, and the 25 compounds had π-π interactions with the residue Phe392 (Table S3). According to the -CDOCKER_ENERGY value and interactions of key residues, 5 intersecting compounds were selected from the 25 compounds for further exploration.

**Figure 2:**
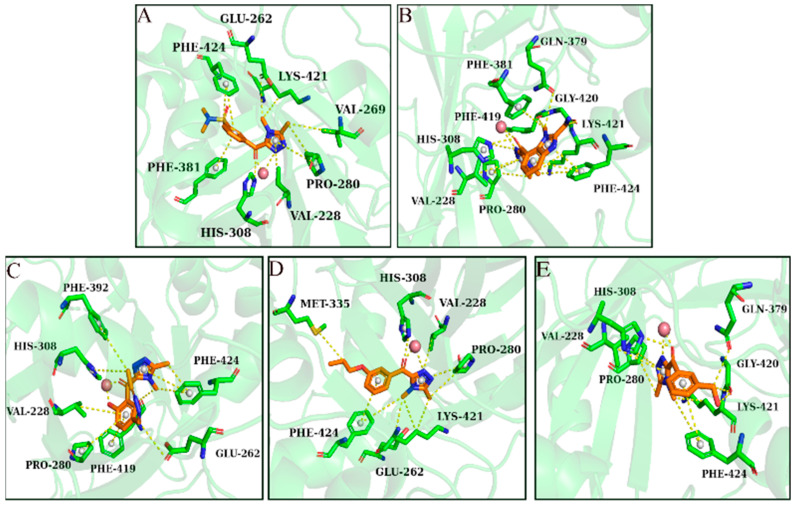
Ligand interaction with the amino residues of HPPD. (**A**): Compound **5**; (**B**): Compound **8**; (**C**): Compound **10**; (**D**): Compound **11**; and (**E**): Compound **25**.

As shown in Figure 2, five ligands formed chelation with the metal Co(Ⅱ) in HPPD, and π-π accumulation with Phe424. As shown in Figure 2A–C, the benzene rings and methyl in the ligands combined with Phe381 through π-π interactions and π–Alkyl interactions. His308 interacted with the carbonyl oxygen atom in the ligand through hydrogen bonding. In addition to the key residues, Pro280 and Val228 formed hydrophobic interactions with the ligands, improving the binding stability between the ligand and protein.

**Figure 3:**
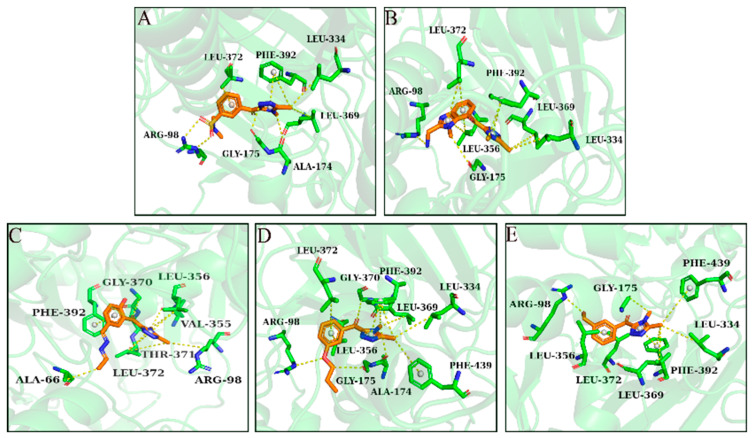
Ligand interaction with the amino residues of PPO. (**A**): Compound **5**; (**B**): Compound **8**; (**C**): Compound **10**; (**D**): Compound **11**; and (**E**): Compound **25**.

As shown in Figure 3, Phe392, Arg98, and Leu372 were the main residues in the PPO enzyme. Phe392 and ligands mainly formed π-π interactions and π–Alkyl interactions. For Arg98, as shown in Figure 3A,D,E, the oxygen atom of the ether bond and hydroxyl in the ligands played a key role in forming hydrogen bonds with Arg98. Leu372 bounded to ligands primarily through π–Alkyl interactions. Leu334 played a role in ligand–receptor binding. All compounds showed high -CDOCKER_ENERGY scores (Table 1). The scoring values of several ligands were higher than that of the original ligand.

### 2.2. Generation and Verification of NBC Model

**Figure 4:**
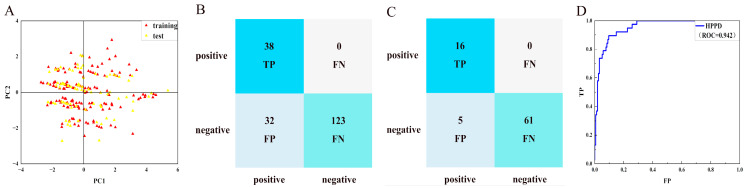
NBC-HPPD model. (**A**): PCA; (**B**): confusion matrix of the NBC model to distinguish the training set; (**C**): confusion matrix of NBC model to distinguish the test sets; and (**D**): ROC curve of the NBC model.

**Figure 5:**
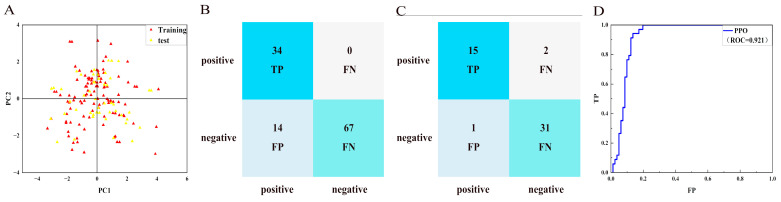
NBC-PPO model. (**A**): PCA; (**B**): confusion matrix of the NBC model to distinguish the training set; (**C**): confusion matrix of the NBC model to distinguish the test sets; and (**D**): ROC curve of the NBC model.

The chemical space diversity of compounds in the training set and the test set affected the performance of the classification model. Principal component analysis (PCA) was performed for the molecular descriptors (Log *p*, Molecular Weight, Num_H_Donors, Num_H_Acceptors and Num_Rotatable) of all compounds. As shown in Figure 4A, the training set (Figure S1A) and test set (Figure S1B) of HPPD inhibitors were widely distributed over a spatial range, indicating the chemical diversity of the selected compounds. In the ML model, the data visualization tool confusion matrix was employed to evaluate the accuracy of the model. As described in Figure 4B,C, the number of false positive (FP) compounds in the HPPD training set was 32 and the number of false negative (FN) compounds was 0. The number of FPs in the test set was five and the number of false negatives was zero. According to the matrix, the constructed model showed a good classification effect. SP, SE, MCC, and ROC values were used to evaluate the model, and the predictive performance of the model was confirmed. As shown in Figure 4D, the ROC value of NBC-HPPD was >0.90 (0.94). In Figure 5A, the PPO training set (Figure S2A) and test set (Figure S2B) have a wide spatial distribution, which confirms the chemical diversity of the compounds utilized in the model construction. For the PPO analysis, as shown in Figure 5B,C, the number of false positives in the training set was 14 and the number of FNs was 0. The number of FPs in the test set was one and the number of FNs was two, which indicated that the model had a good ability to distinguish between active and inactive inhibitors. As shown in Figure 5D, the ROC value of NBC-PPO reached 0.92.

As shown in Table 2, the sensitivity (SE) and specificity (SP) values of the NBC-HPPD model were higher, and the model was capable of identifying active and inactive molecules, with an MCC score of 0.66; in the test set, the model also indicated good predictive performance (ROC: 0.98), with an MCC score > 0.80. NBC-PPO was similar to NBC-HPPD; the ROC values of both the training set and test set were > 0.90, while the SE and SP values were higher. The MCC scores were 0.77 and 0.86, respectively. According to the evaluation indicators, NBC-PPO could effectively identify active and inactive inhibitors.

### 2.3. Virtual Screening via Ligand Similarity Search

Five new compounds were used as templates, a structural similarity search was conducted in the database (280,000 compounds), and 158 similar compounds were obtained. To prevent drug development failure due to unreasonable pharmacokinetics, ADMET (Absorption, Distribution, Metabolism, Excretion, and Toxicity) prediction was performed for the 158 compounds. As shown in Figure S3, most of the compounds were within the 95% confidence interval, meeting the toxicological requirements.

The obtained 158 compounds were submitted to CDOCKER molecular docking with HPPD and PPO (Table S4). The RMSD values between HPPD, PPO, and the natural ligand were 1.385 and 0.892, indicating that the CDOCKER procedure was reliable. The docking values of the two targets were evaluated using the NBC model. There were eight cross-compounds in the two targets that met the requirements. Z-1, Z-2, Z-3, and Z-5 had a stronger affinity for HPPD and PPO. For HPPD, the -CDOCKER ENERGY values of the four compounds were 59.64 kcal/mol, 69.41 kcal/mol, 45.86 kcal/mol, and 58.32 kcal/mol, respectively, and the docking scores were higher than those of the original ligand. For PPO, the -CDOCKER ENERGY values were 34.63 kcal/mol, 34.90 kcal/mol, 35.57 kcal/mol, and 37.40 kcal/mol, respectively. All compounds showed favorable docking results (Table 3).

**Figure 6:**
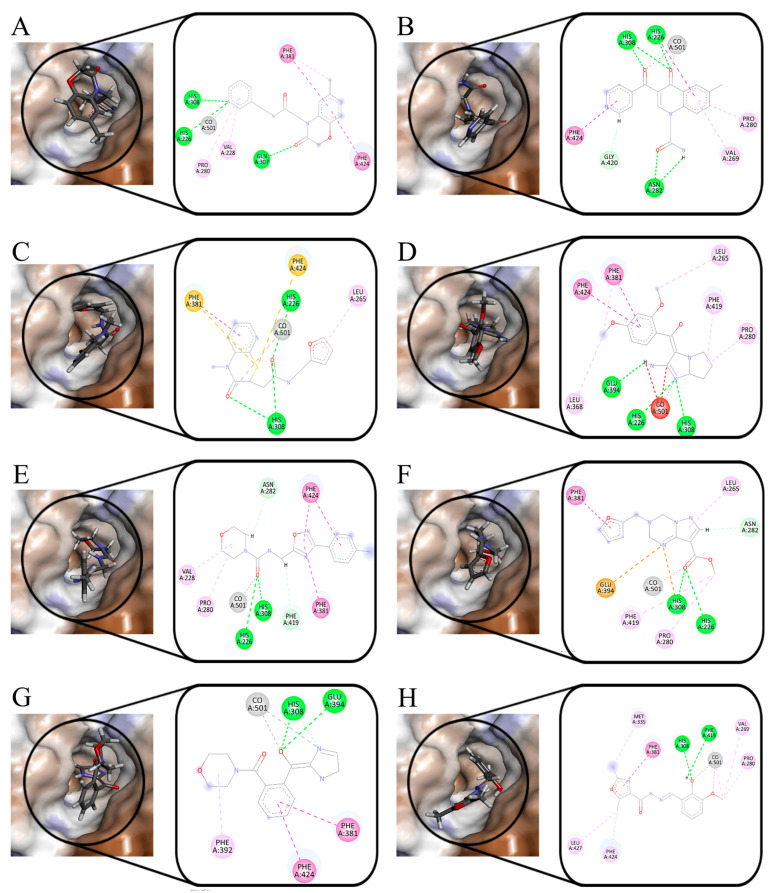
Molecular docking results of the obtained ligands in the active pocket of HPPD. (**A**): Z-1; (**B**): Z-2; (**C**): Z-3; (**D**): Z-4; (**E**): Z-5; (**F**): Z-6; (**G**): Z-7; and (**H**): Z-8.

Figure 6 shows the interaction of eight compounds in the HPPD active pocket. Phe381 and Phe424 were the key residues in the binding process, which formed π-π interactions with the benzene rings of Z-1, Z-4, Z-6, and Z-7. Unlike other compounds, the sulfur atom in Z-3 formed π–sulfur interaction with the two residues. Co (II) in the protein formed coordination with eight compounds. Z-2, Z-3, Z-7, and Z-8 formed a bidentate combination with Co (II). All compounds formed hydrogen bond interaction with His308. His226 formed hydrogen bond interactions with the nitrogen atom in the Z-1 pyridine ring and Z-4 five-membered ring. For the compounds Z-2, Z-3, Z-5, and Z-6, His226 formed hydrogen bond interactions with oxygen atoms in the carbonyl group. In addition to the key residues, there were hydrophobic interactions between the compounds and other residues surrounding the binding pocket. According to the cavity diagram, eight compounds completely embedded inside the cavity.

**Figure 7:**
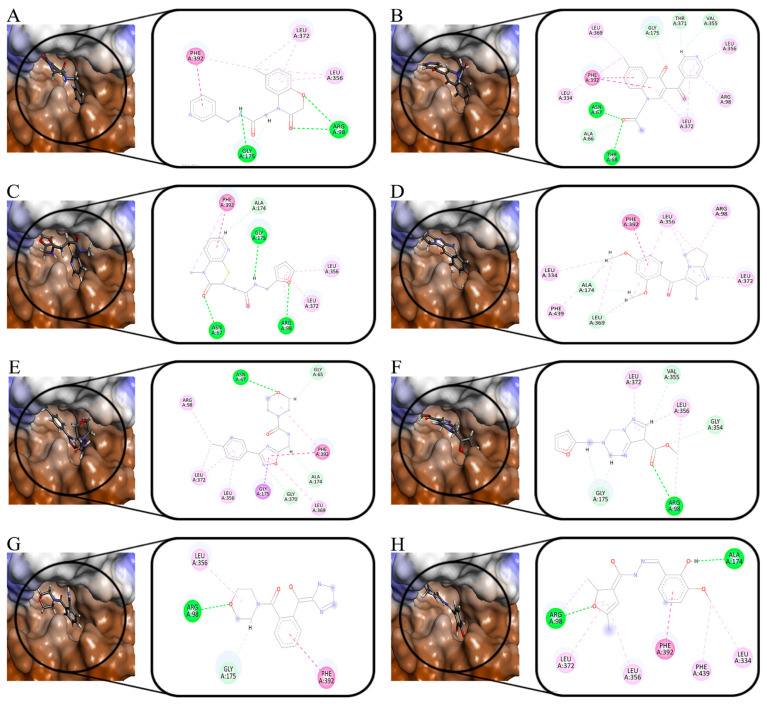
Molecular docking results of the obtained ligands in the active pocket of PPO. (**A**): Z-1; (**B**): Z-2; (**C**): Z-3; (**D**): Z-4; (**E**): Z-5; (**F**): Z-6; (**G**): Z-7; and (**H**): Z-8.

The docking results of PPO are shown in Figure 7. Except for Z-6, Phe392 formed π-π interactions with the benzene ring. The oxygen atom of carbonyl in Z-1 and Z-6 formed hydrogen bond interactions with Arg98, the oxygen atom in the Z-3 and Z-8 oxole ring combined with Arg98 with the same force. For Z-7, the oxygen atoms in the six-membered heterocyclic rings generated interactions with Arg98. Leu356 and Leu372 formed hydrophobic interactions with all compounds. According to the cavity diagram, all the compounds were completely accommodated in the PPO protein cavity.

### 2.4. MD Simulation

**Figure 8:**
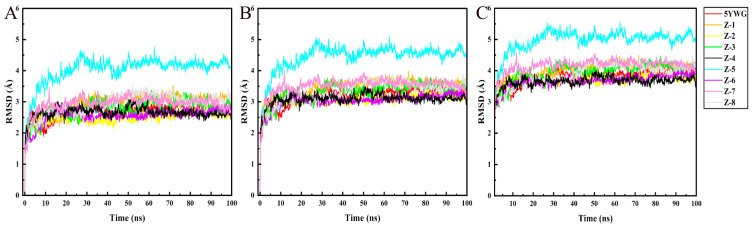
MD simulation results of HPPD and ligands. (**A**) HPPD-RMSD of skeleton Cα atom. (**B**) RMSD of the heavy atom of the ligands; and (**C**) RMSD of the protein active pocket with 5 Å residues around the ligands.

**Figure 9:**
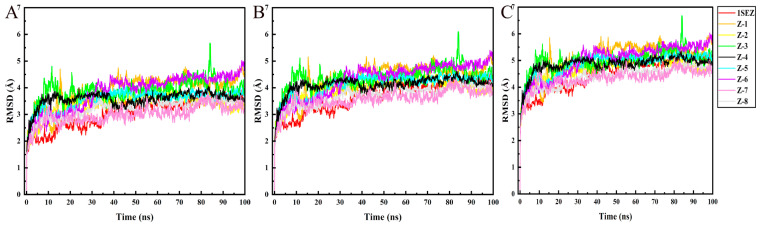
MD simulation results of PPO and ligands. (**A**) PPO-RMSD of skeleton Cα atom. (**B**) RMSD of the heavy atom of the ligands; and (**C**) RMSD of the protein active pocket with 5 Å residues around the ligands.

The accuracy of molecular docking was verified by MD simulation. The simulation time was set to 100 ns, and the stability of the system was evaluated according to the RMSD. The RMSD value of the ligands and *Arabidopsis thaliana* HPPD (*At*HPPD*)* protein is shown in Figure 8. In the MD simulation, the RMSD values of compounds fluctuated in the range of 2–3 Å. All compounds tended to equilibrium after 20 ns of simulation. Except compound Z-5, the RMSD values of seven compounds were similar; the RMSD values of compounds Z-2, Z-4, and Z-6 were slightly lower than that of the natural ligand. According to the Cα atom analysis of the protein backbone, the RMSD values did not fluctuate widely after equilibrium, and the result showed that the screened compounds were stable in binding with the receptor. The RMSD values of the eight compounds and the natural ligand were compared with the PPO protein, and it was found that all the compounds tended to equilibrium after 30 ns (Figure 9). The RMSD value of compound Z-4 was the most stable, and compounds Z-7 and Z-8 were a little bit lower than the natural ligand. The RMSD values of compounds Z-1, Z-3, and Z-6 were similar to the natural ligand.

**Figure 10:**
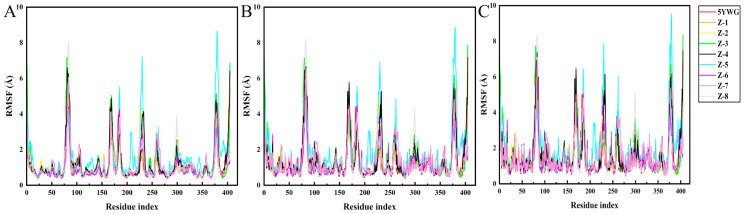
Fluctuations of residues involved in HPPD interactions with ligands. (**A**) HPPD-RMSF of Cα atoms; (**B**) RMSF of the heavy atom of the ligands; and (**C**) RMSF of the protein active pocket with 5 Å residues around the ligands.

**Figure 11:**
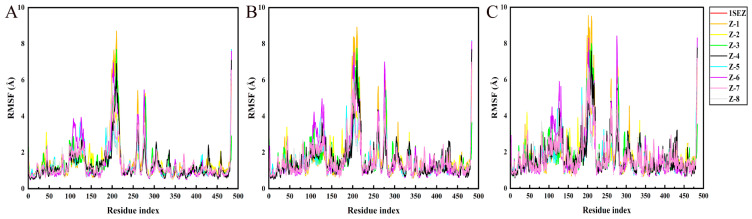
Fluctuations of residues involved in PPO interactions with ligands. (**A**) PPO-RMSF of Cα atoms; (**B**) RMSF of the heavy atom of the ligands; and (**C**) RMSF of the protein active pocket with 5 Å residues around the ligands.

During the simulation, the RMSF values describe the flexibility of each residue in the protein, and higher RMSF values indicate greater residue volatility. As shown in Figure 10, it was observed that the HPPD N-terminal and C-terminal tails fluctuated more than the other parts, and the target protein displayed great fluctuations in the range of residue numbers 160–200, 250–275, and 370–390, which was considered as flexible. For the PPO protein (Figure 11), there were large fluctuations in the 100–140 and 175–225 range; this part was considered as the flexible region.

**Figure 12:**
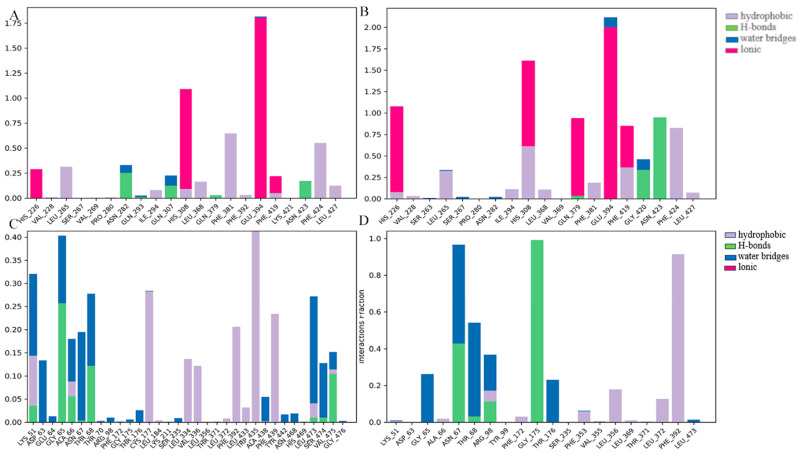
Residue contribution degree (**A**) HPPD: Z-4; (**B**) HPPD: Z-7; (**C**) PPO: Z-4; and (**D**) PPO: Z-7.

As for HPPD (Figure 12), in the MD simulation process, residues Phe424, Phe381, and His308 contributed significantly to the binding process of compounds Z-4 and Z-7 to proteins, and residues Glu394 and His226 played a certain role in the binding interaction. With respect to PPO, residues Gly65, Asn67, Thr68, Arg98, and Phe392 played a key role in the binding of both compounds to proteins. In subsequent studies, the structure may be further optimized by improving the binding of the compound to the residues that contribute more significantly.

The receptor–ligand binding free energy (Δ*G_bind_*) was calculated using the MM/GBSA method. Δ*G_bind_* was evaluated by Δ*G_bind_ Coulomb,* Δ*G_bind_ Covalent,* Δ*G_bind_ Hbond,* Δ*G_bind_ Lipo*, and Δ*G_bind_ vdW*. As shown in Table 4, for HPPD targets, Δ*G_bind_ Coulomb*, Δ*G_bind_ Lipo*, and Δ*G_bind_ vdW* contributed significantly to Δ*G_bind_*. The MM/GBSA of the eight compounds ranged within -40.00–20.00 kcal/mol, the Δ*G_bind_* of compounds Z-1, Z-2, Z-5, Z-6, and Z-7 was < −30 kcal/mol, and the calculated values were −36.91 kcal/mol, −34.73 kcal/mol, −33.13 kcal/mol, −39.40 kcal/mol, and −39.66 kcal/mol, respectively. According to the Δ*G_bind_ Hbond* value, Z-2 and Z-4 produced stable hydrogen bonds with the amino acid residues of the protein, with the values of −1.65 kcal/mol and −1.15 kcal/mol. The molecular docking results confirmed that the two compounds formed hydrogen bond interactions with His308 and His226, which were consistent with MM/GBSA.

MM/GBSA was employed to learn the interaction between medicine and biomolecules; through the analysis of ligands binding to the PPO target, it was found (Table 5) that Δ*G_bind_ Lipo* and Δ*G_bind_ vdW* were the main contributors, and the Δ*G_bind_ Coulomb* values of some compounds were positive, which exerted a negative effect on protein binding. The Δ*G_bind_* values of compounds Z-2, Z-3, Z-5, and Z-8 were −58.94 kcal/mol, −55.10 kcal/mol, −57.61 kcal/mol, and −55.53 kcal/mol, respectively.

### 2.5. HPPD and PPO Enzyme Activities In Vitro and Herbicidal Activity

In order to verify whether the screened compounds exhibited inhibitory effects on HPPD and PPO, the enzyme activity and herbicidal activity of compounds Z-4 and Z-7 were studied. Mesotrione and oxyfluorfen were used as positive controls, and the inhibitory effects of the compounds on HPPD and PPO are listed in Table 6. The IC_50_ values of compound Z-4 on HPPD and PPO were 1.607 μM and 2.932 μM, respectively, which showed obvious inhibitory effects on both targets. The IC_50_ value of compound Z-7 for HPPD was 1.494 μM, which was similar to that of mesotrione (0.904 μM), and the IC_50_ value of compound Z-7 for PPO was 4.232 μM, which was not as good as that of oxyfluofen (0.726 μM). Although compound Z-7 did not inhibit PPO well, it also exhibited a certain inhibitory effect. It can also be considered a potential candidate for HPPD&PPO dual-target inhibitors.

**Figure 13:**
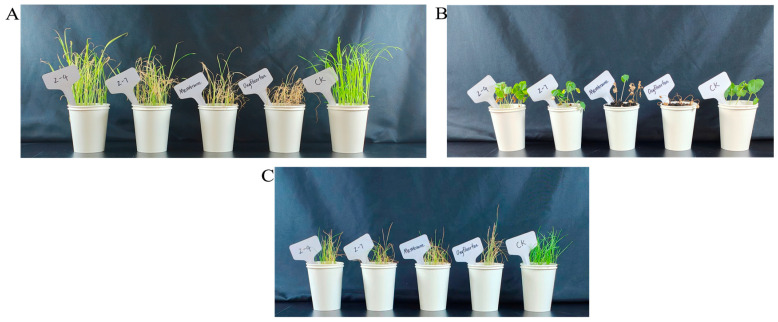
Herbicidal activities of compounds Z-4, Z-7, mesotrione, and oxflurane (post-emergence, 150 g ai/ha, treated after 7 days). (**A**) *EC*. (**B**) *AT*. (**C**) *LP*.

The herbicidal activities on *Echinochloa crus-galli* (*EC*)*, Lolium perenne* (*LP*), and *Abutilon theophrasti* (*AT*) of mesotrione, oxyfluofen, and compounds Z-4 and Z-7 were determined at a dose of 150 g ai/ha. As shown in Figure 13A, *EC* exhibited obvious death after being treated with the positive control compounds mesotrione and oxyfluorfen; the leaves of *EC* treated with Z-4 and Z-7 were also wilted, but the weed control effect was not as good as that of the control. For *AT* (Figure 13B), compounds Z-4 and Z-7 did not exhibit a significant herbicidal effect. In Figure 13C, the two compounds exerted certain inhibitory effects on *LP*, and the inhibition effect of compound Z-7 on weeds was consistent with that of oxyfluofen. The results show that there is a certain application prospect for compounds Z-4 and Z-7 in weed management.

The druggability and safety of compounds are critical factors in drug development. To ensure that Z-4 and Z-7 exhibited herbicidal effects with minimal harm to humans and the environment, ADMET prediction was conducted for the compounds. The results indicated that Z-4 (−3.494) and Z-7 (−2.175) possessed favorable solubility. Regarding CYP2D6 inhibition, the prediction results were “false”, suggesting that these compounds could avoid abnormal blood concentration changes caused by CYP2D6 inhibition. Additionally, both compounds demonstrated low toxicity and favorable environmental profiles.

### 2.6. Results of Reverse Synthesis Analysis

**Figure 14:**
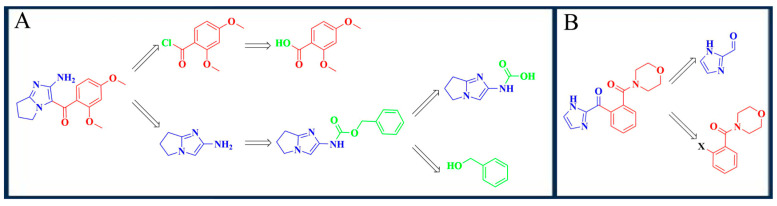
Reverse synthesis routes. (**A**) Z-4 inverse synthetic route; (**B**) Z-7 inverse synthetic route.

Compared with commercial HPPD and PPO inhibitors, it was found that the selected compounds shared similar physical and chemical properties to commercial herbicides. According to the computer simulation and experimental verification, Z-4 and Z-7 were selected to predict the retrosynthesis. The design of the reverse synthesis route was mainly limited by low cost and keeping the reaction steps within three steps. As shown in Figure 14, compound Z-4 was synthesized in three steps and compound Z-7 in one step.

## 3. Materials and Methods

### 3.1. Fragment-Based Drug Design (FBDD) and Molecular Docking

**Figure 15:**
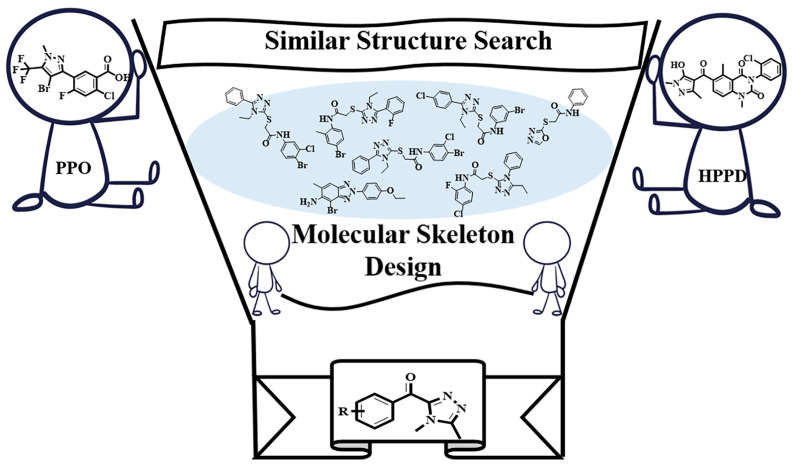
Common skeleton selection.

The original ligands of *At* HPPD (PDB ID: 6LGT) (resolution 1.50 Å) [19] and *Nicotiana tabacum* PPO (*Nt*PPO) (PDB ID:1SEZ) (resolution 2.90 Å) [20] were extracted, and the similar structure of the two natural ligands was searched in a natural compound database based on the structure and fingerprint properties of the compounds. The novel scaffold was finally confirmed (Figure 15). Then, the newly designed skeleton was placed in a binding pocket of two target proteins to construct complex structures and perform skeleton growth. All fragments selected were required follow the “rule of three” (MW < 300, hydrogen bond acceptors ≤3, log *p* ≤ 3) [21,22]. Fragment screening was performed on the fragment library for pesticides of PADFrag (http://chemyang.ccnu.edu.cn/ccb/database/PADFrag/ (accessed on 8 September 2023)) [23] and the Topscience fragment library (https://www.tsbiochem.com) (13,684 fragments).

Discovery Studio (DS) (Biovia Inc., San Diego, CA, USA, 2020) was used to process proteins and set HPPD and PPO binding sites. The receptor protein was pretreated with the “prepare protein” module. The De Novo Link function in the “Receptor-Ligand Interactions” module connected potentially active fragments suitable for binding sites to the skeleton [24]. The “View Interactions” module was selected to examine the interactions between molecules and proteins [25]. The cross-compounds were submitted to the following procedure. The molecular docking experiment employed the “CDOCKER” program under the “Receptor-Ligand Interactions” module of DS software(version 2020) with *At*HPPD and *Nt*PPO*,* respectively. Redundant chains were deleted, water molecules were removed, and the incomplete residues were corrected using the “prepare protein” module. The “Macromolecules” module was used to give the CHARMm force field to the treated proteins [26], the binding site of the protein around the ligand was set to 10 Å, and other settings were default.

### 3.2. Construct and Evaluate Naïve Bayesian Classification (NBC) Models

The NBC model was built based on the fingerprint, number, or text properties of the input compounds to distinguish between active and inactive compounds [27,28]. According to the docking scores of the two targets as identification attributes, the “Create Bayesian Model” module in DS was used to build the NBC model.

For HPPD, a total of 54 active inhibitors and 221 inactive inhibitors were collected. From the data set, 70% of the compounds were randomly selected as the training set and 30% as the test set to construct the NBC model of HPPD. The PPO constructed NBC model was similar to the HPPD model; 51 active inhibitors and 113 inactive inhibitors were collected, and the data set was divided into the training set and the test set in a 7:3 ratio.

In the case of classification methods, ROC values and 5-fold cross-validation were used to evaluate the performance of the model, and true positive (TP), false negative (FN), false positive (FP), true negative (TN), specificity (SP), sensitivity (SE), and Matthews’s correlation coefficient (MCC) for the target were used to evaluate the model [27,29,30]. An MCC higher than 0.4 indicates that the model has predictive performance [31]. The parameters SP, SE, and MCC were calculated as Equations (1)–(3).

(1) $$\mathrm{Sensitivity}=\frac{\mathrm{TP}}{\mathrm{TP}+\mathrm{FN}}$$

(2) $$\mathrm{Specificity}=\frac{\mathrm{TN}}{\mathrm{FP}+\mathrm{TN}}$$

(3) $$\mathrm{MCC}=\frac{\mathrm{TP}\times \mathrm{TN}\;-\;\mathrm{FP}\times \mathrm{FN}}{\surd (\mathrm{TP}+\mathrm{FP})(\mathrm{TP}+\mathrm{FN})(\mathrm{TN}+\mathrm{FP})(\mathrm{TN}+\mathrm{FN})}$$

### 3.3. Virtual Screening for Ligand Similarity Search

Ligand similarity search is one of the virtual screening techniques, which is based on the chemical structure and physicochemical properties of known compounds [32,33]. The “Select Subset Libraries” tool under the DS “Small Molecule” module was used to perform a similarity search for compounds [29]. Scaffold growth with five new compounds was used as a reference, and the compounds’ numeric properties and a fingerprint search protocol were used to query the Chemdiv database (https://www.chemdiv.com/cn/ (accessed on 21 April 2024)). The selected compounds were virtually screened for HPPD and PPO targets, and the cross-compounds produced by the two targets were analyzed. -CDOCKER Energy was selected to score the docking posture of the molecules, and the top 10 conformations of docking procedures were reserved for analysis. The interaction between the compound and the amino residue were analyzed, and the final binding mode of the receptor and ligand was determined.

### 3.4. MD Simulation and Binding Free Energy Calculation

The MD simulation methods evaluate fluctuations and conformational changes in ligand–protein binding over hypothetical time horizons [34]. After the compound was docked with the protein, the complex structure was saved, and MD simulation was carried out in the Desmond module of Schrödinger software (version 12.9). The dynamics simulation system was a simple point charge (SPC) water model, and appropriate counterbalance ion neutralization was added to ensure that the simulation system was neutral. The OPLS_2005 force field was used to minimize the energy of the complex system, the maximum interaction was set to 2000, and the convergence threshold was set to 1.0 kcal mol^−1^ A^−1^ [26,35]. The simulation duration was set to 100 ns, the final generated trajectory report was processed, and the root mean square deviation (RMSD) and root mean square fluctuation (RMSF) data were generated for the protein skeleton, residues around the ligand and binding pockets, and ligand weight atoms. The binding stability of small molecules to receptors was determined.

Binding free energy (Δ*G_bind_*) is an important parameter to describe the affinity of a protein and ligand [36]. After the MD simulation study, the Desmond module of the Schrodinger software (version 12.9) package was used to calculate the MM/GBSA of the compound, and the binding between the compound and protein was reflected in the *ΔG_bind_* result. During the calculation, the OPLS_2005 position and VSGB solvent model were used, and other parameters were the default value [26]. The following equation was to calculate the change in binding free energy:

Δ*G_bind_* = *G_complex_* − (*G_protein_* + *G_ligand_*)

*G_complex_* is the free energy of the complex; *G_protein_* is the free energy of the receptor; and *G_ligand_* is the free energy of the ligand.

### 3.5. AtHPPD and PPO Inhibition Experiment and Herbicidal Activity In Vitro

The compounds Z-4 and Z-7 were purchased from Shanghai Omer Biotechnology Co., Ltd., Shanghai, China. Prior to the in vitro assay, *At*HPPD was prepared and purified as shown in Supporting Information S1. The enzyme inhibitory activity of *At*HPPD in vitro was evaluated using the coupled method. Mesotrione was selected as the positive control and different concentrations of the compounds were configured. The reaction was conducted in HEPES buffer at 30 °C, and the formation of maleylacetoacetate was monitored at 318 nm by UV/vis plate. The inhibition rate at different concentrations was calculated according to the linear relationship between the compound concentration and the inhibition rate, and the IC_50_ value was calculated using SPSS 20.0 software. The PPO enzyme inhibitory activity in vivo was determined using a PPO ELISA kit. After *EC* reached the 2-leaf stage, the leaf tissue was treated according to the instructions of the kit, the absorbance values of different concentrations of each compound were determined at a 450 nm wavelength with ethoxyflurfen as the positive control; the calculation method was the same as for HPPD.

The herbicidal activity of compounds Z-4 and Z-7 was evaluated by using the weeds *EC* and *LP* and the broad-leaved weed *AT* as test subjects. The weeds were grown in a culture chamber (temperature 27 °C, humidity 60%). When the weeds grew to the 1–2 leaf stage, the herbicidal activity test was carried out at a dose of 150 g ai/ha, with mesotrione and oxyfluofen as the positive controls. On the 7th day after treatment, the damage and growth status of the weeds were observed.

### 3.6. Reverse Synthesis Method

Reverse synthesis is an important technique in organic drug synthesis. The reverse synthesis routes of compounds Z-4 and Z-7 were designed using the Scifinder (https://scifinder.cas.org (accessed on 30 July 2024)) database.

## 4. Conclusions

In this study, a variety of virtual screening combinations were used to discover novel inhibitors. Dual-target inhibitors of HPPD&PPO were designed using the FBDD method. According to the interactions of key residues, compounds 5, 8, 10, 11, and 25 were selected as reference templates for a structural similarity search. A total of 158 compounds meeting the criteria were obtained, and the obtained compounds were submitted to CDOCKER for molecular docking with HPPD and PPO. The NBC model was used to evaluate the accuracy of the screening results, and eight cross-compounds conformed to the requirements for the two targets. The MD simulation and MM/GBSA of the eight compounds were studied. Compounds Z-4 and Z-7 exhibited inhibitory effects on HPPD and PPO enzymes in vitro, among which Z-4 was better than Z-7. The compounds showed a certain herbicidal effect on *EC* and *LP* at a 150 g ai/ha dose. Based on the enzyme inhibition and herbicidal activity, the screened compounds have potential to be PPO&HPPD dual-target inhibitors, which can be further developed.

## Figures and Tables

**Table 1:** Molecular docking values of compounds with HPPD and PPO.

Sequence	Structure	-CDOCKER ENERGY-HPPD (kcal/mol)	-CDOCKER ENERGY-PPO (kcal/mol)
natural ligand	/	25.65	4.722
Compound **5**	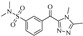	25.98	18.24
Compound **8**	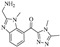	21.20	12.34
Compound **10**	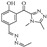	24.35	15.65
Compound **11**	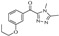	31.93	11.29
Compound **25**	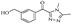	30.01	14.28

**Table 2:** Evaluation indicators of NBC-HPPD and NBC-PPO.

Models	ROC Score	ROC Rating	SE	SP	MCC
HPPD (training set)	0.94	Excellent	1.00	0.79	0.66
HPPD (test set)	0.98	Excellent	1.00	0.92	0.84
PPO (training set)	0.92	Excellent	1.00	0.83	0.77
PPO (test set)	0.96	Excellent	0.88	0.97	0.86

**Table 3:** Docking score of HPPD and PPO.

Name	Structure	-CDOCKER ENERGY-HPPD (kcal/mol)	-CDOCKER ENERGY-PPO (kcal/mol)
natural ligand	/	42.04	4.72
Z-1	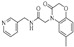	59.64	34.63
Z-2	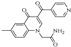	69.41	34.90
Z-3	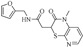	45.86	35.57
Z-4	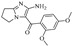	25.40	10.20
Z-5	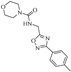	58.32	37.40
Z-6	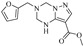	48.79	31.39
Z-7	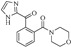	26.26	16.48
Z-8	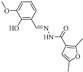	44.54	31.43

**Table 4:** HPPD contribution of various energy components to binding free energy (kcal mol^−1^).

Compound	Δ*G_bind_*	Δ*G_bind_ Coulomb*	Δ*G_bind_ Covalent*	Δ*G_bind_ Hbond*	Δ*G_bind_ Lipo*	Δ*G_bind_ vdW*
Z-1	−36.91	−12.53	4.55	−0.75	−18.92	−44.86
Z-2	−34.73	−34.40	9.92	−1.65	−19.61	−42.88
Z-3	−24.94	−22.71	5.191	−0.68	−16.33	−40.32
Z-4	−20.50	−23.72	12.58	−1.15	−15.55	−38.51
Z-5	−33.13	−14.67	8.83	−0.03	−17.47	−35.81
Z-6	−28.49	−24.05	4.94	−0.44	−13.78	−35.43
Z-7	−39.40	−31.50	3.16	−0.99	−14.86	−35.36
Z-8	−39.66	−36.73	6.02	−0.57	−18.71	−37.43

**Table 5:** PPO contribution of various energy components to binding free energy (kcal mol^−1^).

Compound	Δ*G_bind_*	Δ*G_bind_ Coulomb*	Δ*G_bind_ Covalent*	Δ*G_bind_ Hbond*	Δ*G_bind_ Lipo*	Δ*G_bind_ vdW*
Z-1	−49.84	−20.31	2.76	−1.54	−17.89	−40.42
Z-2	−58.94	−15.02	5.23	−1.28	−18.88	−51.46
Z-3	−55.10	−27.19	3.87	−2.05	−20.16	−45.03
Z-4	−44.89	8.97	5.78	−0.15	−18.61	−49.66
Z-5	−57.61	−7.89	6.61	−0.55	−21.20	−53.81
Z-6	−40.96	5.09	4.33	−0.06	−20.02	−41.90
Z-7	−41.93	−26.29	9.25	−1.59	−18.06	−37.22
Z-8	−55.53	0.15	4.41	−0.81	−21.33	−45.27

**Table 6:** Inhibitory activities of the compounds against HPPD and PPO.

Compound	IC_50_ (μM)	
	*At*HPPD	PPO
Mesotrione	0.904	-
Oxyfluofen	-	0.726
Z-4	1.607	2.932
Z-7	1.494	4.232

## Data Availability

The original contributions presented in this study are included in the article/Supplementary Materials. Further inquiries can be directed to the corresponding author.

## Supplementary Materials

The following supporting information can be downloaded at: https://www.mdpi.com/article/10.3390/molecules30071491/s1, Table S1: Physicochemical properties of novel compounds; Table S2: Interaction of compounds with HPPD residues; Table S3: Interaction of compounds with PPO residues; Table S4. The structure and evaluation of the potential compounds; Figure S1: HPPD for constructing Bayesian model A: Training set; B: Test set; Figure S2: PPO for constructing Bayesian model A: training set; B: Test set; Figure S3: ADMET diagram of the compound.
