# Supplementary material for: Virtual Screening and Bioassay of Novel Protoporphyrinogen Oxidase and p-Hydroxyphenylpyruvate Dioxygenase Dual-Target Inhibitors
Source: Molecules. 2025 Mar 27;30(7):1491. doi: 10.3390/molecules30071491 (PMC11990379; doi:10.3390/molecules30071491)
Supplement: Supplementary file 1 [file molecules-30-01491-s001.zip › molecules-3526219-supplementary.pdf]

Support information

Virtual screening and bioassay of novel protoporphyrinogen oxidase & p-hydroxyphenylpyruvate dioxygenase dual-target inhibitors

Panxiu Zhang[1,†], Haifeng Cao[1,2,†], Tiansong Li[1] and Ying Fu[1,3,*]

[1] Department of Chemistry, College of Arts and Sciences, Northeast Agricultural University, Harbin 150030, China

[2] National Soybean Engineering Technology Research Center, Northeast Agricultural University, Harbin 150030, China

[3] Key Laboratory of Agricultural Functional Molecule Design and Utilization of Heilongjiang Province, Harbin 150030, China

[†]These two authors contributed equally to this work

[*]Corresponding Author: Ying Fu, Email address: fuying@neau.edu.cn

Csupport information S1 ....S-1

Table S1 Physicochemical properties of novel compounds S-3

Table S2 Interaction of compounds with HPPD residues...S-4

Table S3 Interaction of compounds with PPO residues..S-5

Table S4 The structure and evaluation of the potential compounds..S-17

Figure S1 HPPD for constructing Bayesian model A: training set; B: Test set.................................S-21

Figure S2 PPO for constructing Bayesian model A: training set; B: Test set....................................S-24

Figure S3 ADMET diagram of the compound..S-25

Csupport information S1

Recombinant *At*HPPD was overexpressed in Escherichia coli BL21 cells using pET-15b-HPPD plasmid. The culture was grown overnight in Luria-Bertani broth containing 100μg/ml Amp at 37 °C. When OD_{600} reached 0.4, the cells were induced with 0.1 mM isopropyl β-D-1-thiogalactopyranoside (IPTG) and incubated with HPPD at 16 °C for 16 hours. The bacteria were obtained by centrifugation (4 °C, 5000 rpm,10 min), washed and suspended with buffer solution (20 mM HEPES, 20 mM NaCl, pH 7.0). The supernatant was obtained by ultrasonic treatment with cell crusher and centrifugation at 13000 rpm.

Affinity purification of supernatant was performed. The flow rate was set and the protein was eluted with 15% buffer (20 mM HEPES, 150 mM NaCl, 500 mM imidazole, pH 7.0) and 60% buffer (20 mM HEPES, 150 mM NaCl, 500 mM imidazole, pH 7.0). Target proteins were collected. AtHPPD is concentrated using a centrifugal filter (Millipore) and stored at -80 °C.

Table S1. Physicochemical properties of novel compounds

Sequence	Structure	MW	HBDs	HBAs	ARs	Log *p*
1	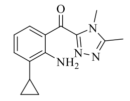	256.303	1	3	3	1.547
2	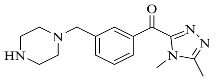	299.371	1	5	4	0.966
3	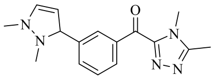	297.355	0	4	3	0.640
4	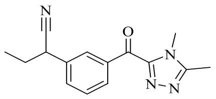	268.314	0	4	4	2.303
5	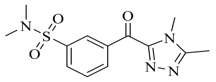	308.356	0	5	4	0.588
6	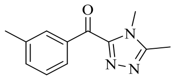	215.251	0	3	2	1.957
7	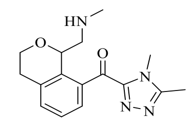	300.356	1	5	4	0.981
8	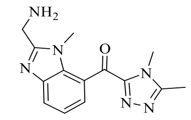	284.316	1	5	3	0.492
9	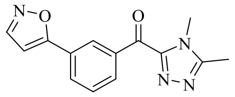	268.271	0	4	3	1.735
10	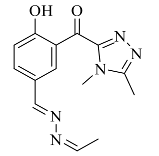	285.301	1	6	4	1.122
11	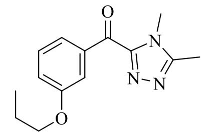	259.304	0	4	5	2.327
12	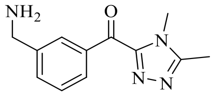	230.266	1	4	3	0.577
13	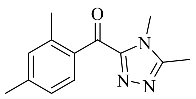	229.278	0	3	2	2.443
14	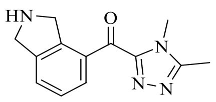	242.276	1	4	2	0.762

15	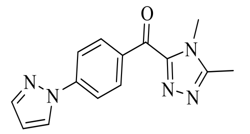	267.286	0	4	3	1.697
16	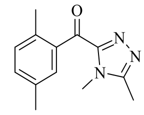	229.278	0	3	2	2.443
17	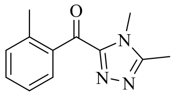	215.251	0	3	2	1.957
18	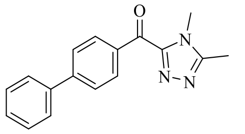	277.32	0	3	3	2.989
19	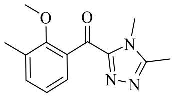	245.277	0	4	3	1.941
20	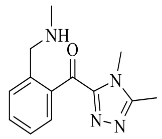	244.292	1	4	4	1.009
21	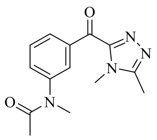	272.302	0	4	3	0.797
22	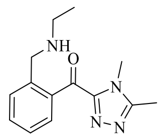	258.319	1	4	5	1.358
23	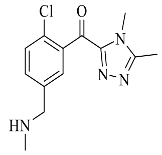	278.737	1	4	4	1.673
24	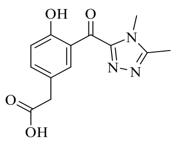	275.26	2	6	4	0.894
25	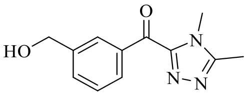	231.251	1	4	3	0.866

Table S2. Interaction of compounds with HPPD residues

Sequence	-CDOCKER ENERGY	Phe424	Phe381	Phe392	Phe419	Gln293	HIS308
1	5.663	Pi-Alkyl	/	/	Pi-Lone pair	/	Hydrogen Bond
2	9.816	Hydrogen Bond	Pi-Sigma	Pi-Pi	Pi-Alkyl	/	Hydrogen Bond
3	1.293	/	Pi-Alkyl	Pi-Pi	Pi-Alkyl	/	Hydrogen Bond
4	25.998	Pi-Pi	Pi-Pi	/	/	/	Hydrogen Bond
5	25.980	Pi-Pi	Pi-Pi	/	/	/	Hydrogen Bond
6	12.425	Pi-Alkyl	/	/	Π-Lonepair	/	Hydrogen Bond
7	18.042	Pi-Pi	Pi-Alkyl	/	Pi-Alkyl	/	Hydrogen Bond
8	21.200	Pi-Pi	Pi- Alkyl	/	Hydrogen Bond	/	Hydrogen Bond
9	23.704	Pi-Pi	/	/	Pi-Alkyl	Hydrogen Bond	Hydrogen Bond
10	24.351	Pi-Pi	/	Pi-Alkyl	Hydrogen Bond	/	Hydrogen Bond
11	31.934	Pi-Pi	/	/	/	/	Hydrogen Bond
12	23.243	Pi-Pi	/	Pi-Alkyl	Hydrogen Bond	Hydrogen Bond	/
13	9.556	/	Pi-Pi	Pi-Alkyl	Pi-Alkyl	Pi-Alkyl	Hydrogen Bond
14	17.835	Pi-Alkyl	Pi-Pi	/	Hydrogen Bond	/	Hydrogen Bond
15	4.528	/	Pi-Pi	/	Pi-Alkyl	/	Hydrogen Bond
16	10.254	/	Pi-Pi	Pi-Alkyl	Pi-Alkyl	Pi-Alkyl	Hydrogen Bond
17	18.577	Pi-Alkyl	/	/	Hydrogen Bond	/	Hydrogen Bond
18	2.548	Pi-Pi	/	/	Pi-Alkyl	/	Hydrogen Bond
19	5.977	Pi-Alkyl	Pi-Pi	/	Hydrogen Bond	/	Hydrogen Bond
20	24.255	Pi-Alkyl	Pi-Pi	/	Hydrogen Bond	/	Hydrogen Bond
21	37.639	Pi-Pi	Pi-Pi	Pi-Alkyl	/	Pi-Alkyl	Hydrogen Bond
22	17.142	Pi-Pi	Pi-Pi	/	Pi-Alkyl	/	Hydrogen Bond
23	19.371	Pi-Pi	Pi-Pi	/	Hydrogen Bond	/	Hydrogen Bond
24	42.551	Pi-Pi	Pi-Pi	Pi-Alkyl	/	/	Hydrogen Bond
25	30.005	Pi-Pi	/	/	/	/	Hydrogen Bond

Table S3. Interaction of compounds with PPO residues

Sequence	-CDOCKER ENERGY	Phe392	Arg98	Leu372	Leu356
1	6.549	Pi-Pi	Pi-Alkyl	Pi-Alkyl	Pi-Alkyl
2	7.725	Pi-Pi	Hydrogen Bond	Pi-Alkyl	Pi-Alkyl
3	1.875	Pi-Pi	Hydrogen Bond	/	/
4	12.014	Pi-Pi	Pi-Alkyl	Pi-Alkyl	Pi-Alkyl
5	18.243	Pi-Pi	Hydrogen Bond	Pi-Sigma	/
6	9.774	Pi-Pi	Pi-Alkyl	Pi-Alkyl	Pi-Alkyl
7	13.379	Pi-Pi	Pi-Alkyl	Pi-Alkyl	Pi-Alkyl
8	12.344	Pi-Pi	Hydrogen Bond	Pi-Alkyl	Pi-Alkyl
9	7.052	Pi-Pi	Pi-Alkyl	Pi-Alkyl	Pi-Alkyl
10	15.652	Pi-Pi	Pi-Alkyl	Pi-Alkyl	Pi-Alkyl
11	11.286	Pi-Pi	Hydrogen Bond	Pi-Alkyl	Pi-Alkyl
12	14.641	Pi-Pi	Pi-Alkyl	Pi-Alkyl	Pi-Alkyl
13	12.944	Pi-Pi	Pi-Alkyl	Pi-Alkyl	Pi-Alkyl
14	2.345	Pi-Pi	Pi-Alkyl	Pi-Alkyl	Pi-Alkyl
15	6.103	Pi-Pi	Pi-Alkyl	Pi-Alkyl	Pi-Alkyl
16	12.714	Pi-Pi	Pi-Alkyl	Pi-Alkyl	Pi-Alkyl
17	10.269	Pi-Pi	Pi-Alkyl	Pi-Alkyl	Pi-Alkyl
18	3.219	Pi-Pi	Pi-Alkyl	Pi-Alkyl	Pi-Alkyl
19	8.665	Pi-Pi	Pi-Alkyl	Pi-Alkyl	Pi-Alkyl
20	11.885	Pi-Pi	Pi-Alkyl	Pi-Alkyl	Pi-Alkyl
21	10.876	Pi-Pi	Pi-Alkyl	Pi-Alkyl	Pi-Alkyl
22	14.484	Pi-Pi	Pi-Alkyl	Pi-Alkyl	Pi-Alkyl
23	11.308	Pi-Pi	Hydrogen Bond	Pi-Alkyl	Pi-Alkyl
24	18.449	Pi-Pi	Hydrogen Bond	Pi-Alkyl	Pi-Alkyl
25	14.281	Pi-Pi	Hydrogen Bond	Pi-Alkyl	Pi-Alkyl

Table S4. The structure and evaluation of the potential compounds

Name	Structure	-CDOCK ENERY (HPPD)	-CDOCK ENERY (PPO)
Compound41829	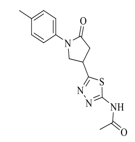	44.195	22.827
Compound74859	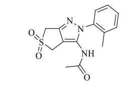	10.651	2.818
Compound35684	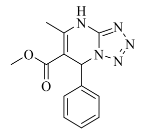	14.721	3.332
Compound70317	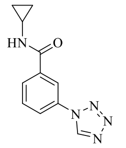	29.406	2.621
Compound80795	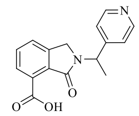	38.752	20.073
Compound66792	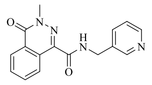	49.364	27.219
Compound51067	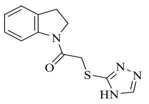	2.631	18.089
Compound72461	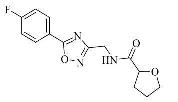	56.464	19.772
Compound35201	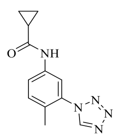	30.865	0.773
Compound77736	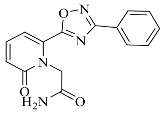	55.440	29.246
Compound86699	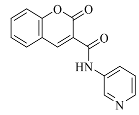	36.005	24.505
Compound61868	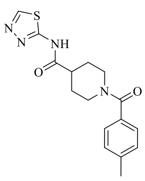	42.119	20.167

Compound40013	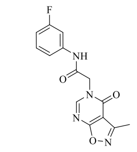	32.726	19.693
Compound66038	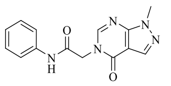	29.313	11.417
Compound16949	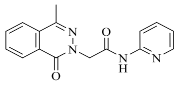	54.020	26.406
Compound73010	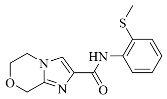	32.707	23.755
Compound41046	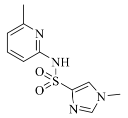	44.513	20.232
Compound42842	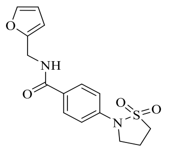	41.620	23.122
Compound74577	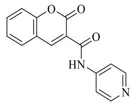	38.577	23.525
Compound15200	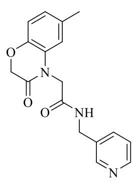	59.637	34.628
Compound38433	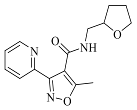	24.760	14.590
Compound38028	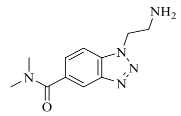	45.351	19.349
Compound1689	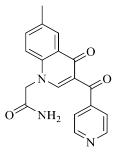	69.414	34.903
Compound73811	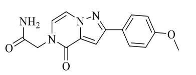	54.630	27.146
Compound64993	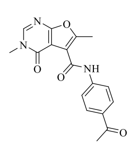	51.791	22.476

Compound31792	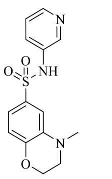	39.943	22.247
Compound45948	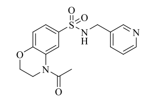	57.248	32.406
Compound42801	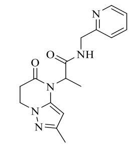	56.468	38.031
Compound52679	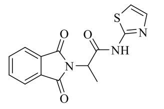	48.752	12.952
Compound66041	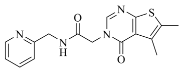	41.809	29.885
Compound32937	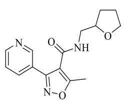	29.358	15.409
Compound73072	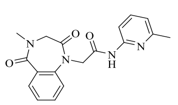	40.916	26.820
Compound34206	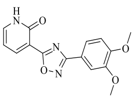	45.487	19.724
Compound39610	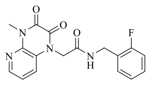	51.004	33.098
Compound19031	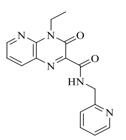	43.566	30.529
Compound33953	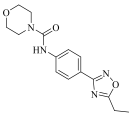	45.954	27.937
Compound2218	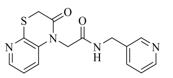	54.934	29.391
Compound42505	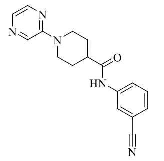	34.285	17.040
Compound15279	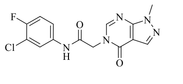	26.763	9.658

Compound15186	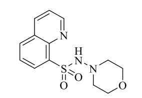	21.054	9.715
Compound49675	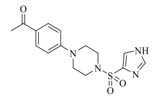	47.077	24.382
Compound78189	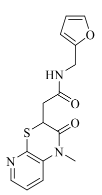	45.857	35.568
Compound42800	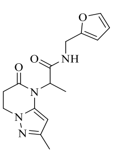	41.947	37.079
Compound32955	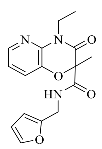	37.731	30.609
Compound14385	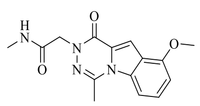	42.116	14.645
Compound27940	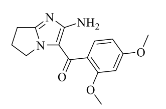	25.402	10.204
Compound18779	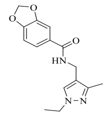	26.750	6.805
Compound41705	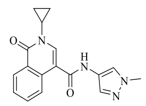	16.853	11.594
Compound4539	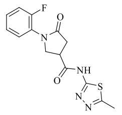	41.762	19.860
Compound19320	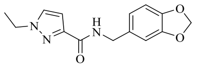	28.445	5.234
Compound59873	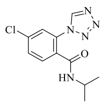	21.989	5.405
Compound7600	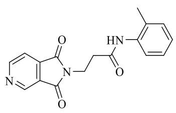	47.710	16.993

Compound4429	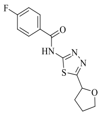	25.053	3.446
Compound75495	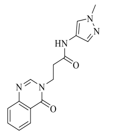	46.406	19.554
Compound49615	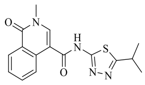	33.835	22.214
Compound84505	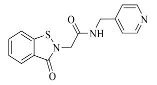	52.158	24.601
Compound87399	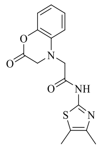	40.932	28.840
Compound75813	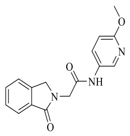	44.449	15.484
Compound12226	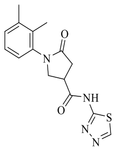	43.433	21.404
Compound11917	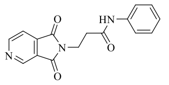	43.379	14.533
Compound81224	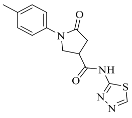	42.325	20.353
Compound63776	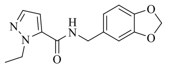	29.635	5.348
Compound68386	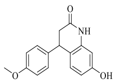	31.416	23.640
Compound43628	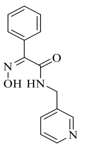	53.221	27.779
Compound24309	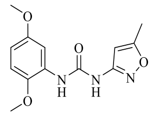	59.041	26.097
Compound11571	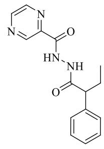	47.393	26.531

Compound13295	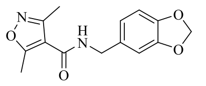	30.328	16.500
Compound38591	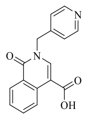	48.882	28.526
Compound30309	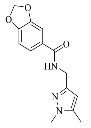	24.300	5.792
Compound83754	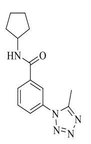	14.796	6.751
Compound3060	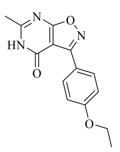	25.170	13.874
Compound33805	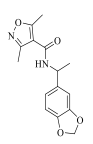	30.221	17.670
Compound30924	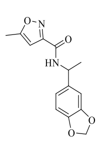	51.067	17.935
Compound82518	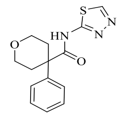	24.548	14.092
Compound17552	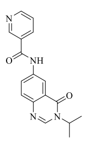	38.500	17.037
Compound1173	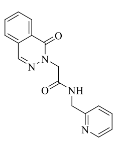	49.251	31.079
Compound47222	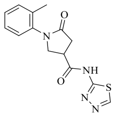	42.321	17.254
Compound86042	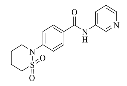	42.852	16.642
Compound68497	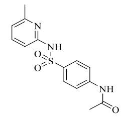	57.743	25.171

Compound39660	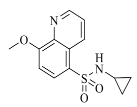	36.038	9.203
Compound38263	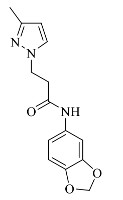	27.804	6.091
Compound9915	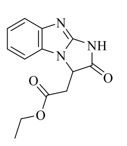	33.571	13.884
Compound53359	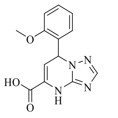	41.227	18.883
Compound16144	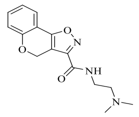	33.083	17.725
Compound22386	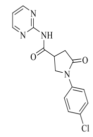	49.493	29.138
Compound54609	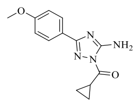	39.584	13.284
Compound19051	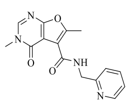	49.656	28.245
Compound69110	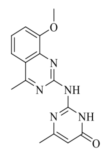	9.8517	14.816
Compound71767	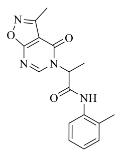	33.833	22.295
Compound87350	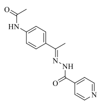	61.832	33.868
Compound77654	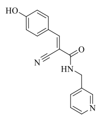	40.609	25.217

Compound47373	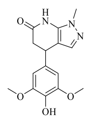	16.687	16.287
Compound60083	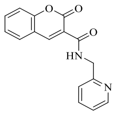	50.886	26.940
Compound35756	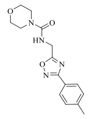	58.319	37.396
Compound31419	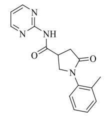	57.327	30.699
Compound56690	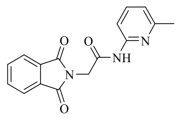	46.915	20.050
Compound54501	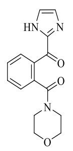	26.263	16.475
Compound19770	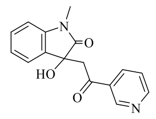	27.370	10.889
Compound60818	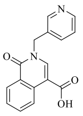	55.322	25.184
Compound56406	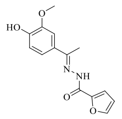	55.903	34.264
102Molecule	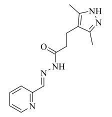	45.868	24.692
103Molecule	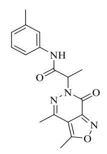	38.527	27.931
104Molecule	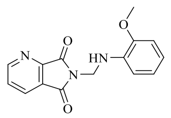	30.780	7.553
105Molecule	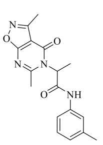	25.770	22.040

106Molecule	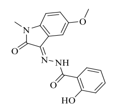	34.701	10.799
107Molecule	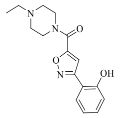	42.343	20.534
108Molecule	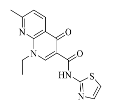	46.896	33.104
109Molecule	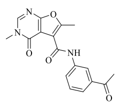	48.408	27.270
110Molecule	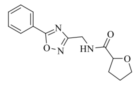	46.012	19.703
111Molecule	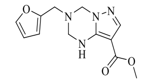	48.788	31.393
112Molecule	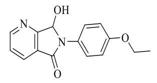	33.431	10.196
113Molecule	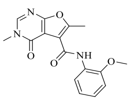	32.717	23.943
114Molecule	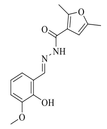	44.541	31.425
115Molecule	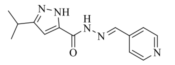	26.117	23.796
116Molecule	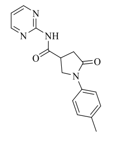	55.576	33.961
117Molecule	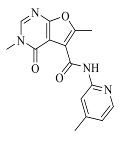	32.713	21.701
118Molecule	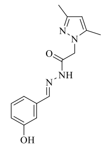	45.174	22.334
119Molecule	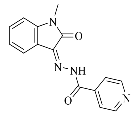	32.644	11.316

120Molecule	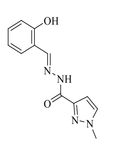	41.383	20.916
121Molecule	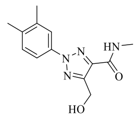	39.403	26.552
122Molecule	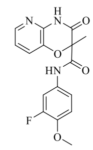	48.738	23.106
Compound143057	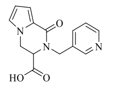	59.557	21.843
Compound87134	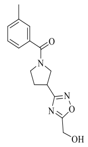	52.434	23.296
Compound82426	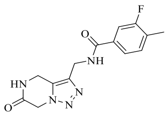	39.299	25.961
Compound78535	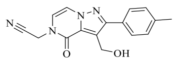	35.401	24.922
Compound68263	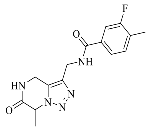	39.659	29.386
Compound77634	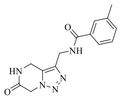	46.967	32.146
Compound32384	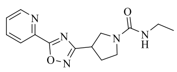	45.999	24.881
Compound13058	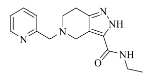	36.723	20.330
Compound143414	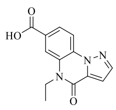	42.338	23.418
Compound50426	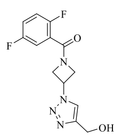	19.458	0.582
Compound135949	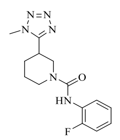	32.451	13.251

Compound141434	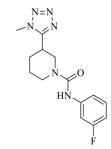	31.452	17.355
Compound143022	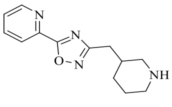	39.415	19.125
Compound50733	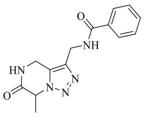	50.372	32.375
Compound79237	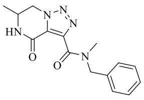	44.026	22.904
Compound143182	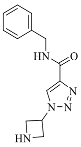	0.476	-10.283
Compound143087	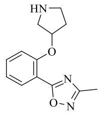	17.068	6.034
Compound90381	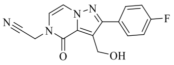	35.677	20.364
Compound16586	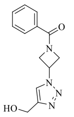	23.128	0.823
Compound11272	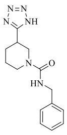	19.712	21.601
Compound6167	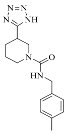	34.177	17.334
Compound3245	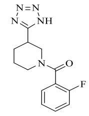	27.440	6.782
Compound141336	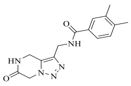	29.051	28.364
Compound40717	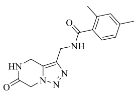	48.397	26.058

Compound61732	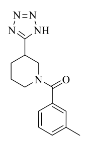	30.345	10.755
Compound36289	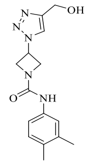	25.651	6.402
Compound72089	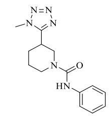	16.733	14.757
Compound128964	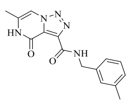	42.804	22.305
Compound2281	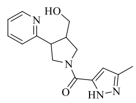	32.302	8.328
Compound17483	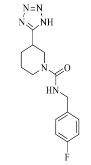	21.307	24.051
Compound128201	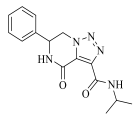	30.396	25.375
Compound38527	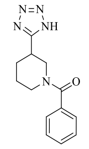	29.917	5.139
Compound121691	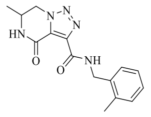	57.200	24.767
Compound142929	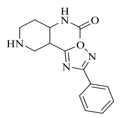	51.821	26.316
Compound24972	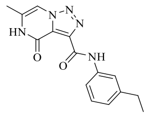	45.234	20.001
Compound143220	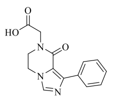	54.285	24.800
Compound106483	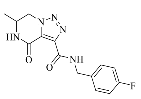	53.813	24.814

A

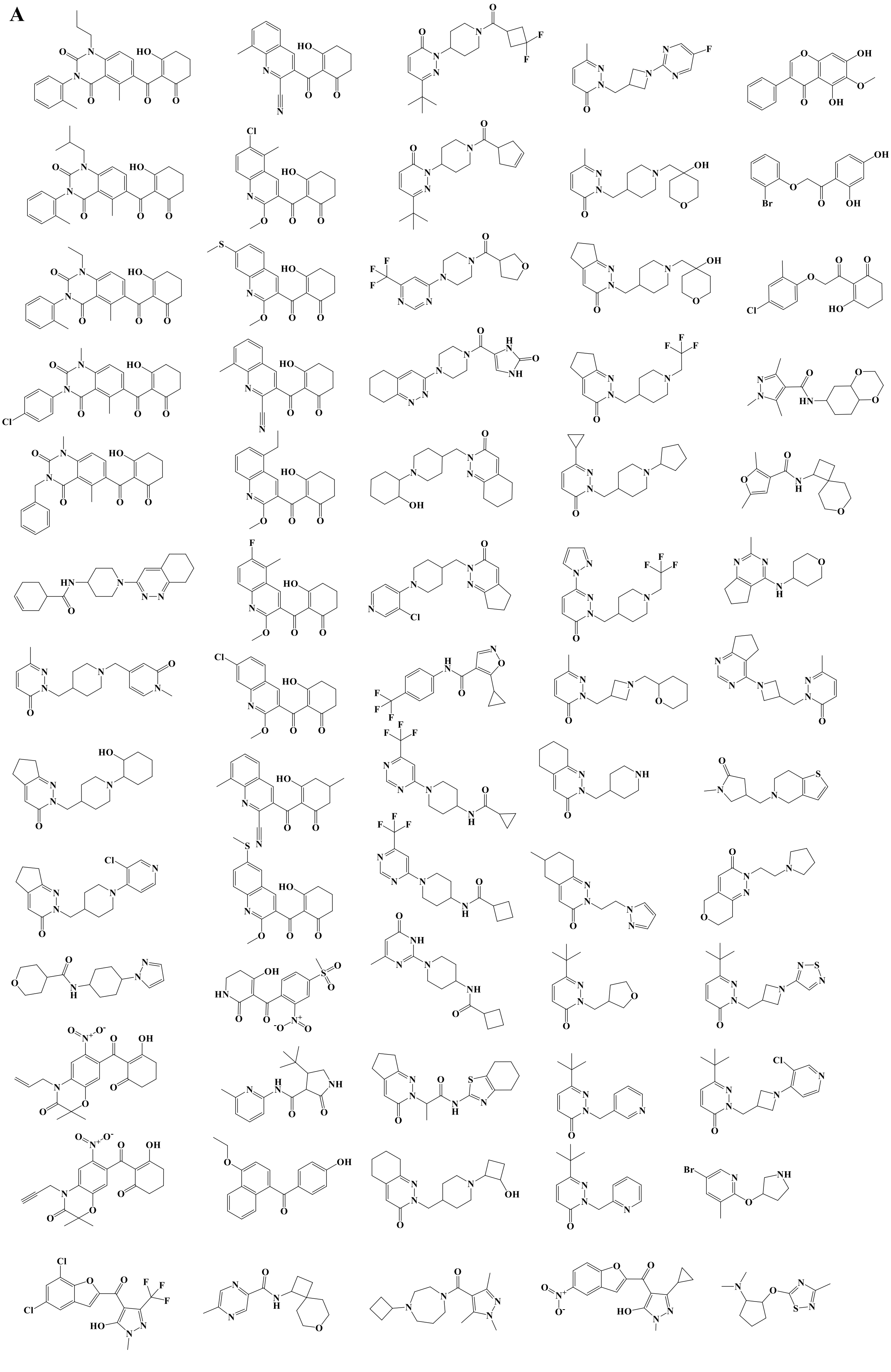

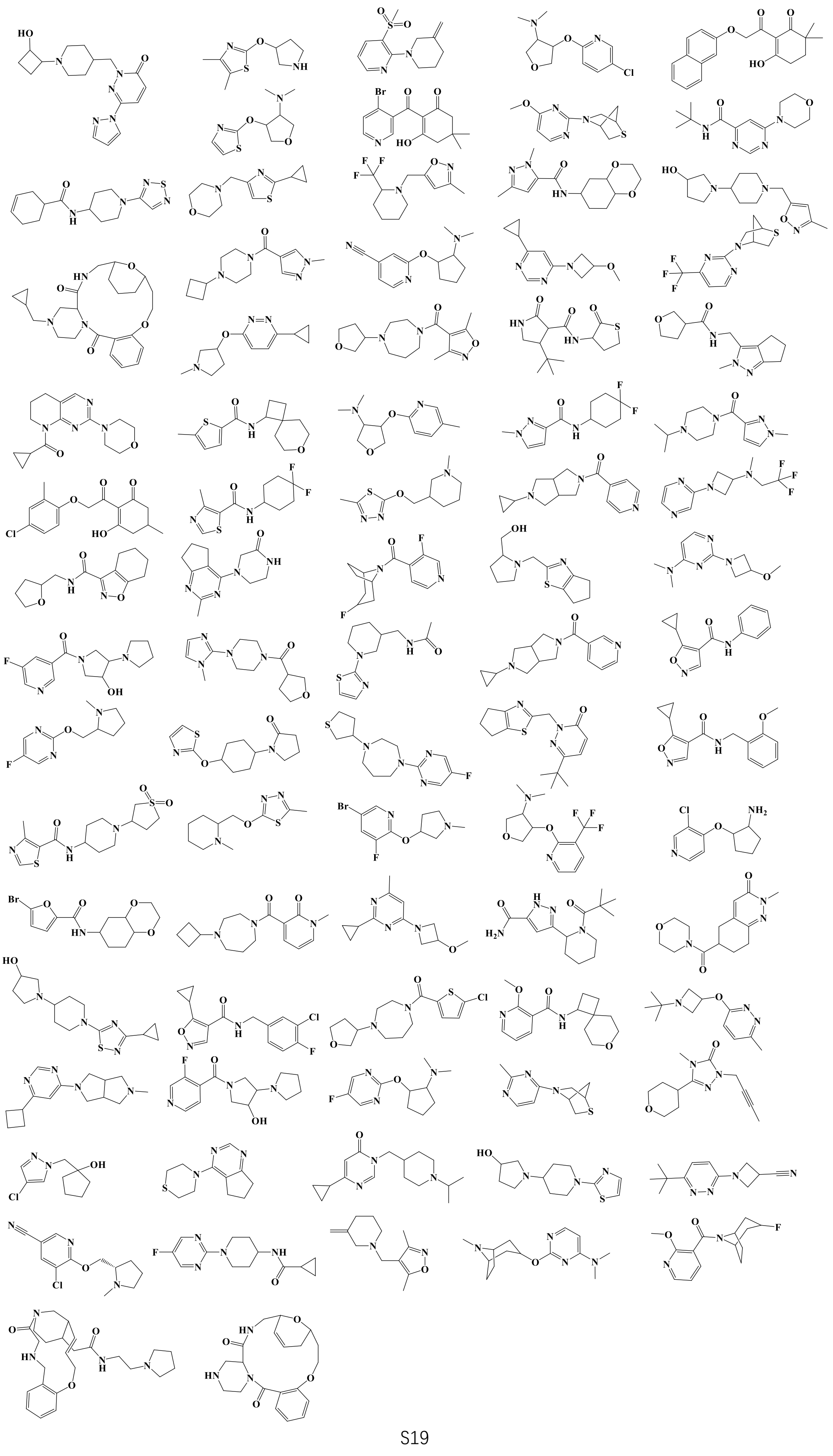

B

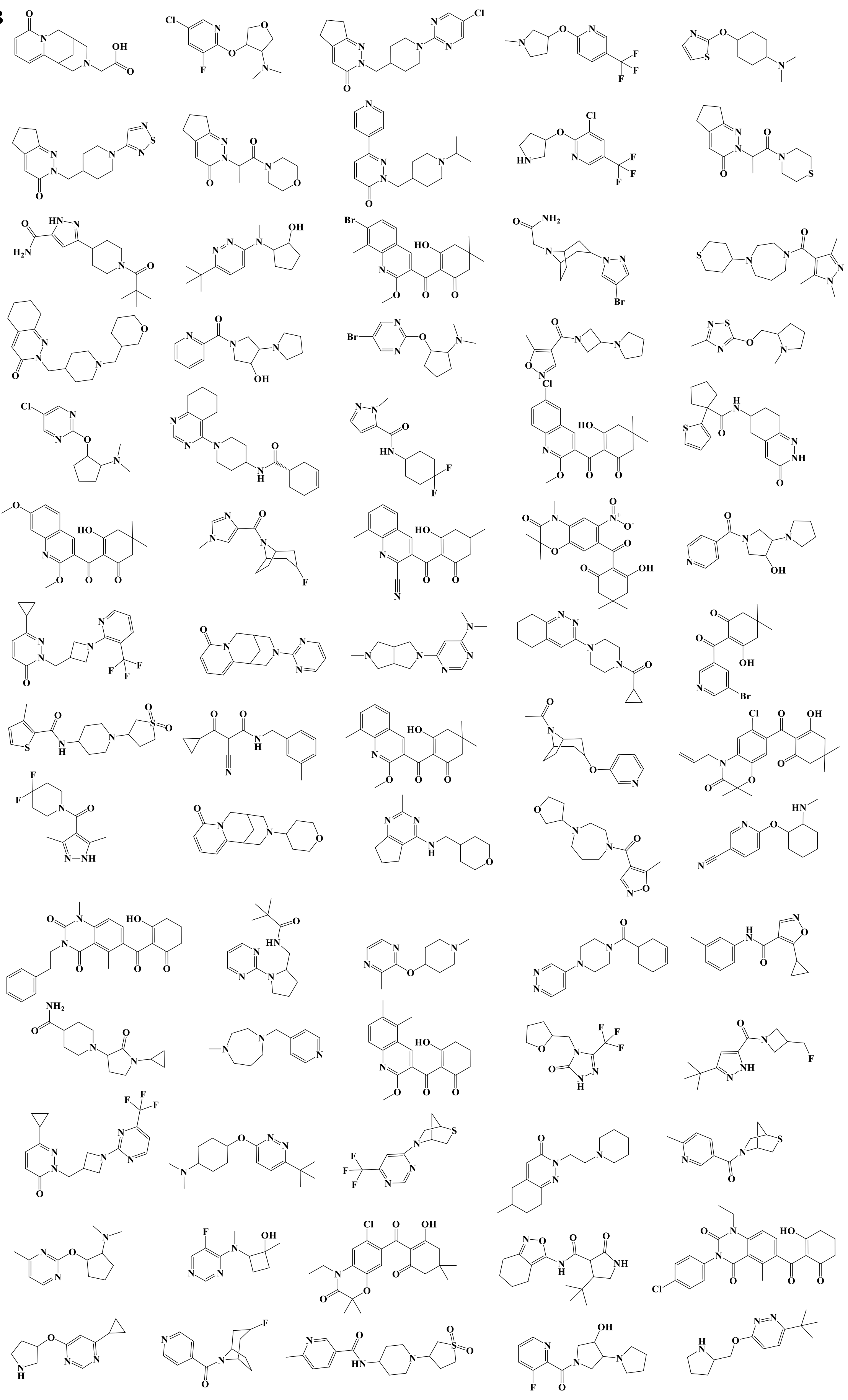

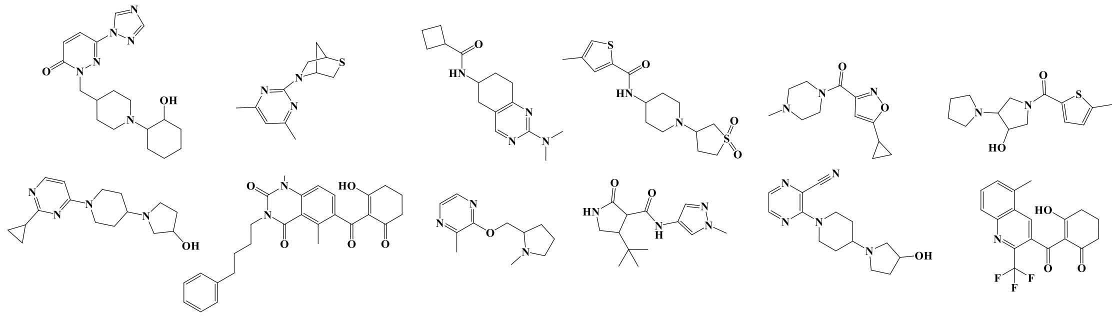

Figure S1. HPPD for constructing Bayesian model A: Training set; B: Test set

A

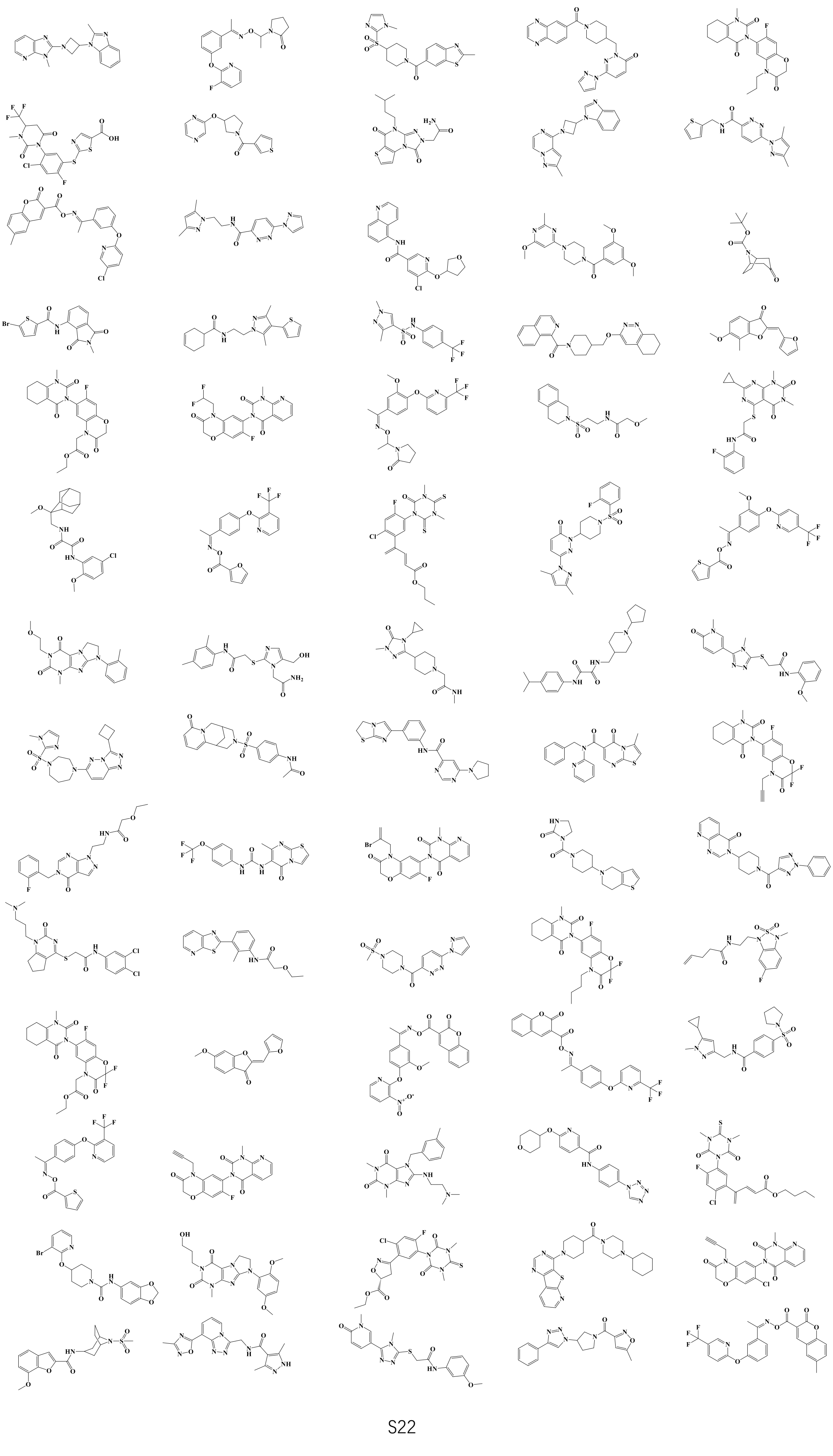

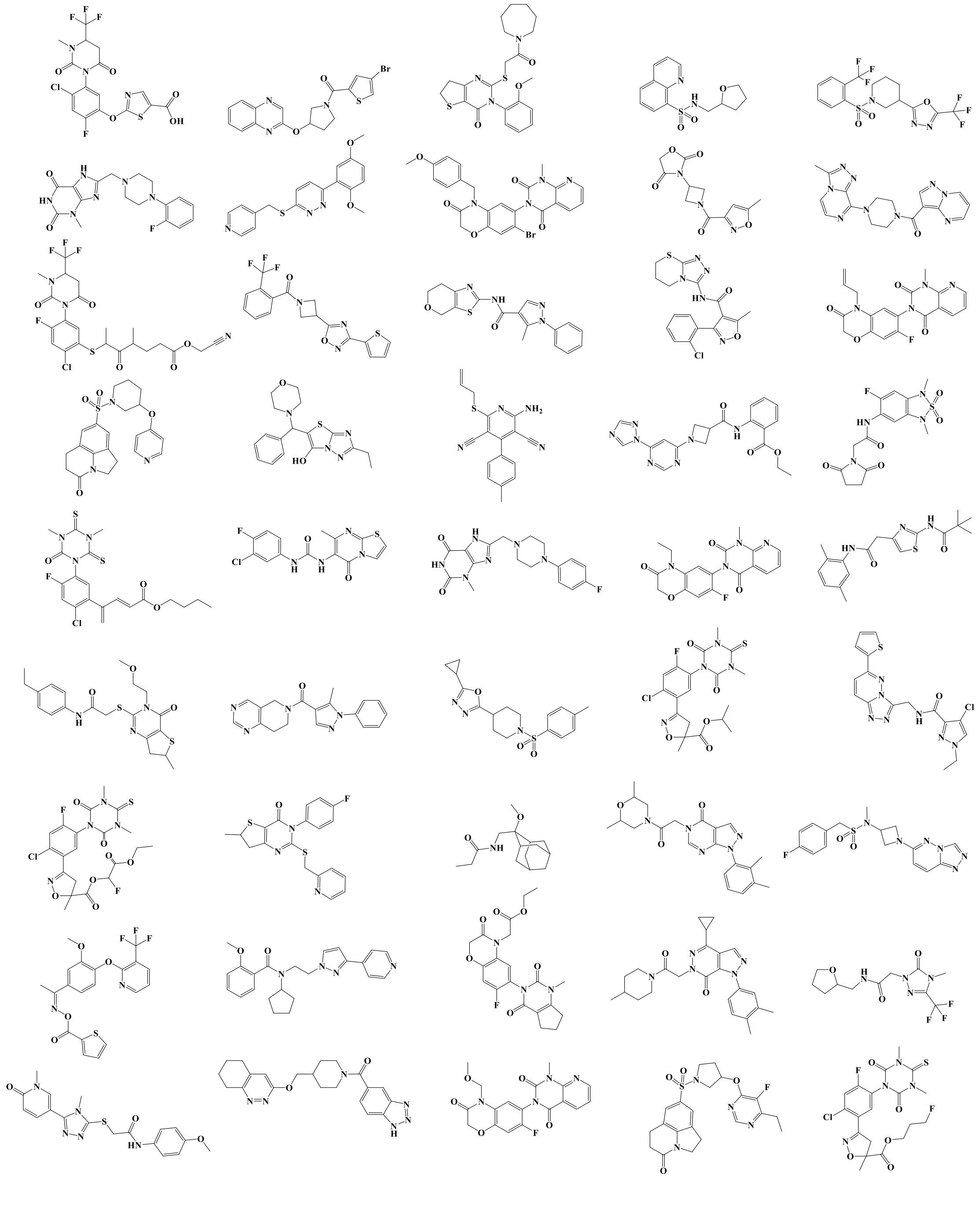

B

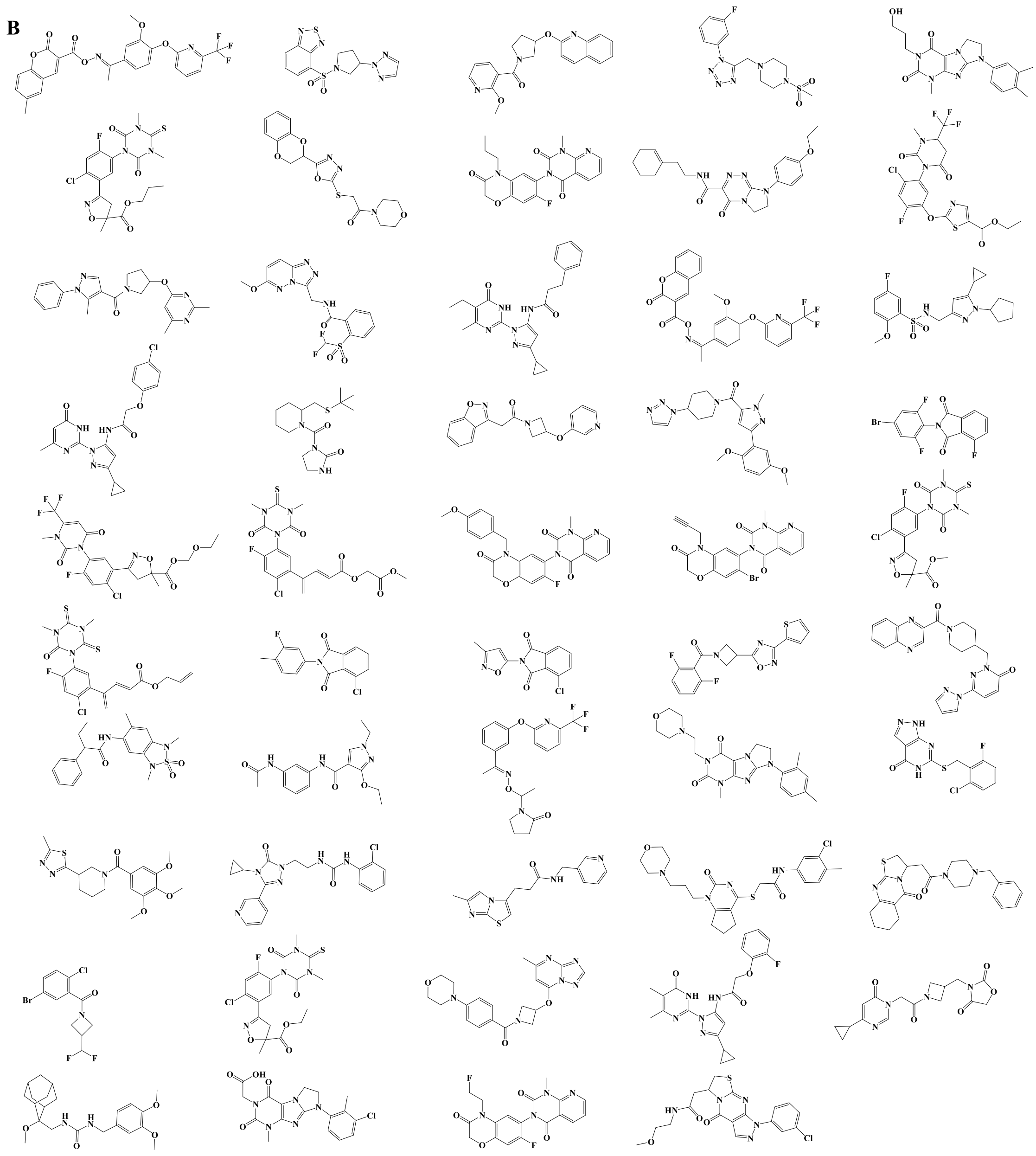

Figure S2. PPO for constructing Bayesian model A: Training set; B: Test set

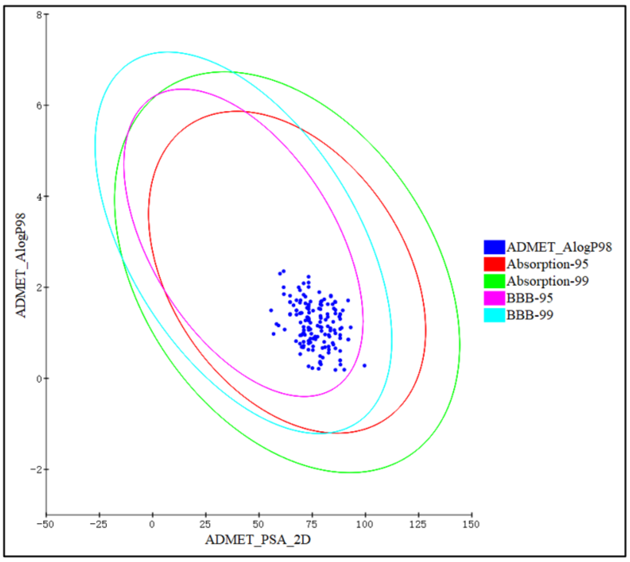

Figure S3. ADMET diagram of the compound
